# Deposition Velocity of PM2.5 in the Winter and Spring above Deciduous and Coniferous Forests in Beijing, China

**DOI:** 10.1371/journal.pone.0097723

**Published:** 2014-05-19

**Authors:** Fengbin Sun, Zhe Yin, Xiaoxiu Lun, Yang Zhao, Renna Li, Fangtian Shi, Xinxiao Yu

**Affiliations:** 1 College of Soil and Water Conservation, Beijing Forestry University, Haidian District, Beijing, China; 2 College of Forestry, Beijing Forestry University, Haidian District, Beijing, China; 3 College of Environmental Engineering, Beijing Forestry University, Haidian District, Beijing, China; 4 College of Environmental Science and Engineering, Peking University, Beijing, China; The Ohio State University, United States of America

## Abstract

To estimate the deposition effect of PM2.5 (particle matter with aerodynamic diameter <2.5 µm) in forests in northern China, we used the gradient method to measure the deposition velocity of PM2.5 during the winter and spring above a deciduous forest in Olympic Forest Park and above a coniferous forest in Jiufeng National Forest Park. Six aerosol samplers were placed on two towers at each site at heights of 9, 12 and 15 m above the ground surface. The sample filters were exchanged every four hours at 6∶00 AM, 10∶00 AM, 2∶00 PM, 6∶00 PM, 10∶00 PM, and 2∶00 AM. The daytime and nighttime deposition velocities in Jiufeng Park and Olympic Park were compared in this study. The February deposition velocities in Jiufeng Park were 1.2±1.3 and 0.7±0.7 cm s^−1^ during the day and night, respectively. The May deposition velocities in Olympic Park were 0.9±0.8 and 0.4±0.5 cm s^−1^ during the day and night, respectively. The May deposition velocities in Jiufeng Park were 1.1±1.2 and 0.6±0.5 cm s^−1^ during the day and night, respectively. The deposition velocities above Jiufeng National Forest Park were higher than those above Olympic Forest Park. The measured values were smaller than the simulated values obtained by the Ruijgrok et al. (1997) and Wesely et al. (1985) models. However, the reproducibility of the Ruijgrok et al. (1997) model was better than that of the Wesely et al. (1985) model. The Hicks et al. (1977) model was used to analyze additional forest parameters to calculate the PM2.5 deposition, which could better reflect the role of the forest in PM2.5 deposition.

## Introduction

Measuring the deposition effect of PM2.5 (particulate matter with aerodynamic diameter <2.5 µm) in forests is an important objective. This study is the first effort to estimate the deposition effect of PM2.5 in forests in northern China. Previous studies have been conducted in Europe and North America [1]–[2] but only one other study has been conducted in Asia. Kazuhide et al. [3] measured the deposition velocity of PM2.5 sulfate in the summer using the gradient method in a deciduous forest at the eastern foot of Mt. Asama, Nagano Prefecture in central Japan. They obtained results that are similar to those measured in other parts of the world. Further investigations of deposition velocities in forests have been conducted in many sites in North America, Europe, Southeast Asia, and East Asia [4]–[6]. However, in East Asia deposition velocities in forests have only been studied in Japan and Chinese Taiwan locations [7]–[15].

The State Forestry Administration of China established this project to study the regulatory function and technology related to forest PM2.5. This study is a major project for exploring the role of the forest in PM2.5 deposition.

Particulate matter 2.5 (PM2.5) is the most important contributor to haze. Under certain conditions, haze can cause the attenuation of atmospheric visibility. The use of motor vehicles in Beijing has significantly increased in recent years: since the 1990s, the vehicle quantity has reached approximately 5.30 million [16]. Furthermore, the total amount of atmospheric pollution in Beijing is increasing, which may be partly due to the heavy use of firecrackers and fireworks on Chinese New Year and other similar occasions. Based on a report from the Ministry of Environmental Protection of China, the PM2.5 concentration reached 1000 µg/m^3^ in Beijing on the Chinese New Year due to the use of 313,000 boxes of fireworks [17]. The Chinese Government has been concerned about air pollution; thus, 35 environmental monitoring stations have been built in Beijing. These stations include 12 urban environmental monitoring stations, 11 suburban environmental monitoring stations, and 7 urban traffic environmental monitoring stations. A total of 24 hours of the measured data from all of these stations was published at http://zx.bjmemc.com.cn in 2013. In this study, we used the gradient method to survey temporal and spatial variations in PM2.5 concentration.

## Experimental Section

### 1. Ethics Statement

This study did not involve any endangered or protected species. This work was conducted based on Forestry Standards “Observation Methodology for Long-term Forest Ecosystem Research” of the People’s Republic of China (LY/T 1952–2011).

### 2. Sites

**Figure 1:**
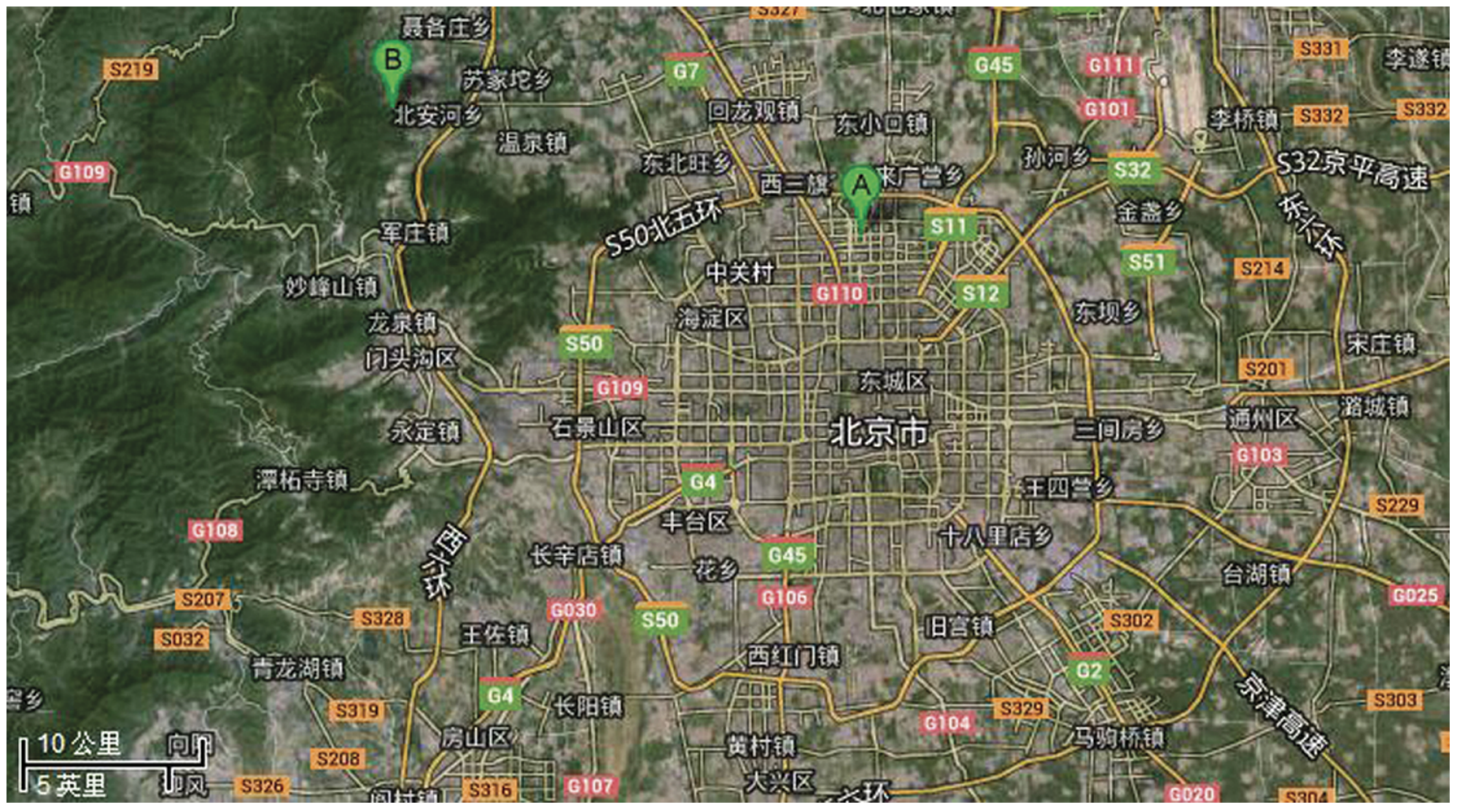
The map of the two fields. A: Olympic Forest Park in Beijing. B: Jiufeng National Forest Park in Beijing.

The two field sites are depicted in Figure 1. One experiment site, Jiufeng National Forestry Park, is managed by the Forestry Committee of Beijing Forestry University and is available for teaching and research use by the university. It is located in Jiufeng National Forest Park in the Beijing Haidian District (40.06°N, 116.09°E). It is a typical rural sampling site.

The other experiment site, Olympic Forest Park, is managed by the Beijing Olympic Forest Park Development and Management Co., Ltd. It is located in a deciduous forest in the Olympic Forest Park in the Beijing Haidian District (40.258°N, 116.39°E). This site represents urban air pollution and is adjacent to Fifth Ring Road, one of the busiest roads in Beijing. *Populus tomentosa* is a winter-deciduous tree and is the dominant species in Olympic Forest Park. The dominant species in the Jiufeng National Forest Park is *Platycladus orientalis* evergreen. This site also contains *Pinus tabulaeformis*. The forests at both sites are classified as temperate forests, and flourish in similar climates.

The height of the trees was approximately 8 m around the tower in both sites. The displacement height ranged approximately from 4 m to 8 m (equations 1, 2 and 3). The roughness length ranged from 0.5 m to 1.5 m during the year for the Olympic and Jiufeng parks (equations 1, 2 and 3). According to measurements at the sites, the leaf area index (*LAI*) at the Jiufeng National Forest Park site was 3.1 and 3.8 in the winter and spring, respectively. The leaf area index (*LAI*) at the Olympic Forest Park site was 1.8 in the spring.

### 3. Sampling Program

**Figure 2:**
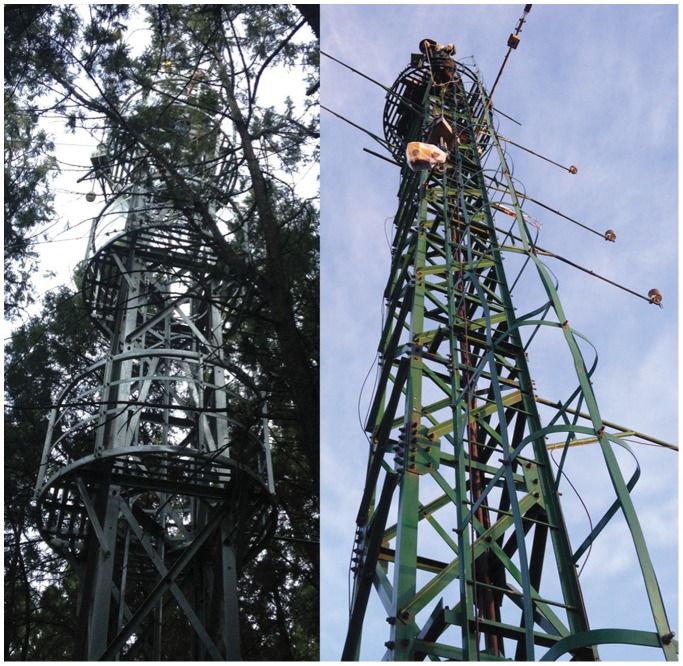
The experiment setup at the study sites. The left panel is the tower in Olympic Forest Park in Beijing and the right panel is the tower in Jiufeng National Forest Park in Beijing.

Two experiment stations were built, one in each forest, with iron towers that measured approximately 16 m in height. Figure 2 depicts the setup of our experiment at both sites.

PM2.5 fluxes were obtained using an atmospheric particulate sampler (KC-6120 integrated air sampler, Qingdao, Laoshan Electronic Instrument Factory Co., Ltd.). PM2.5 was collected on glass fiber filters (MK360, Munktell&Filtrak GmbH, Sweden) at a flow rate of 100 L/min. The samplers were calibrated with the flow meter. Furthermore, the base of the filter film and the cutting head were ultrasonically cleaned with deionized water three times before each experiment.

Three aerosol samplers were placed on each of the two towers at heights of 9, 12 and 15 m above the ground. The sample filters in the atmospheric particulate sampler were changed at 6∶00 AM, 10∶00 AM, 2∶00 PM, 6∶00 PM, 10∶00 PM, and 2∶00 AM (i.e., three times during the day and three times during the night). The ultrasonic anemometer was placed on the iron tower at 15 m, and the meteorological instruments were placed on the iron tower at 9, 12 and 15 m. The sampling times occurred in February 2013 and May 2013. The experiment was performed in the winter from February 22 to 28 and in the spring from May 7 to 12 in 2013. After every sample was obtained, each sample filter was sealed in a clean membrane polypropylene filter box to avoid contamination.

### 4. Meteorological Data and PM10 Data

The meteorological data were measured by meteorological instruments in this study. The measured meteorological data included humidity and temperature (HMP45C, Campbell Scientific Inc., U.S.A.), wind speed and direction (014A/024A, Met One Instruments Inc., U.S.A.). An ultrasonic anemometer (Wind Master, Gill Instruments, Britain) was used to obtain friction velocity and the Monine-Obukhov length.

Some data (PM10 and PM2.5 concentrations) were obtained from the Beijing municipal environmental monitoring center, the agency’s official website is http://zx.bjmemc.com.cn/. The data were applied to calculate the concentration ratios of PM2.5 from PM10. Data is available in Data S1 for this manuscript.

### 5. Computation of PM2.5 Fluxes

In this study, the gradient method was used to estimate PM2.5 fluxes [18]–[20]. Table 1 lists all of the symbols and units used in this research. The equations 1, 2 and 3 were used to determine the *d* and *Z_0_*.
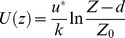
(1)
*u(z)* is the average wind velocity, u* is friction velocity, *k* is Von Karman constant, *d* is roughness length, *Z*_0_ is displacement height.
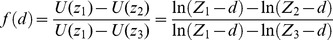
(2)
*U(z*_1_ *)*, *U(z*_2_ *)* and *U(z*_3_ *)* are the wind velocity at heights *Z*_1_, *Z*_2_ and *Z*_3_, respectively. In this study we used 15, 12 and 9 m.
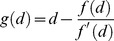
(3)
*f(d)* and *g(d)* are arbitrary unction used in the iterative process and *f'(g)* is the numerical derivative (the gradient between values in two iterations) of *f(d)*. This equation set is used iteratively. First an initial value for *d* is assumed. Then, the value of *g(d)* is updated and used to determine the new value d, then the updated d value is substituted into the equations (2) and (3) to get a new *g(d)*. We assumed the process has converged once the absolute value of the difference between the updated *g(d)* and the previous one is less than 0.001. The final value of *d* = *g(d)*.

**Table 1:** The parameters associated with the research.

F	fluxes	△c	the changes in the concentrations between Z_1_ and Z_3_
u*	friction velocity	C	the PM2.5 concentration at height Z_3_
c*	eddy concentration	D	the zero-plane displacement height
LAI	leaf area index	L	the Monine-Obukhov length
k	Von Karman constant	*Ψ_h_*	the integrated stability correction function
Z_1_	height of 9 m	D	the transfer velocity
Z_2_	height of 12 m	Z_0_	the roughness length
Z_3_	height of 15 m	RH	relative humidity
V_d_	deposition velocity	S_c_	the Schmidt number
P_r_	the Prandtl number	R_s_	the stomatal
R_m_	the mesophyll	R_lu_	the outer surface resistances
R_ac_	resistances to transfer	R_dc_	the resistance to transfer by buoyant convection
R_gs_	resistances to uptake	R_cl_	the resistance to the uptake by exposed surfaces
T	temperature		

The flux-gradient technique was used to determine the flux (*F*) from the measured vertical gradients of the concentration and the eddy diffusivity of sensible heat, as shown in equation (4).
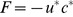
(4)




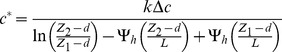
(5)




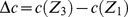
(6)


In this equation, *L* is the Monin-Obukhov length, c^*^ is eddy concentration, Δc is the changes in the concentrations between *Z*_1_ and *Z*_3_. *Ψ_h_* is the integrated stability correction function in atmospheric deposition in relation to acidification and eutrophication per Erisman and Draaijers (1995) [19].
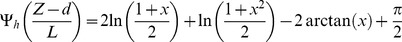
(7)




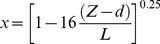
(8)


From equations (4) and (5), *F* can be expressed by equation (9):
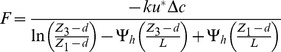
(9)
u* and *L* were averaged every 15 min, *F* was averaged every 4 h because Δ*c* were from six periods: 6∶00–10∶00, 10∶00–14∶00, 14∶00–18∶00, 18∶00–22∶00, 22∶00–2∶00, and 2∶00–6∶00.

The deposition velocity, *V_d_*, was determined using the following equation adapted from Wesely and Hicks (1977) [21]:
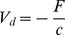
(10)


### 6. Empirical Models

We chose three empirical models with which to compare our measured data: the models proposed by Wesely and Hicks (1977), Wesely et al. (1985), and Ruijgrok et al. (1997) [21], [22], [23]. The model by Wesely et al. (1985) is a dry deposition model over grass, whereas the Ruijgrok et al. (1997) model is a dry deposition model over forest canopy. The Wesely and Hicks (1977) model is a dry deposition model over all canopies.

Slinn (1982) [24] and Wesely (1985) used the aerodynamic terms equation to express the deposition velocities of the aerosol particles. The following equation was used to calculate the deposition velocities, where *R_a_* is the aerodynamic drag, and *V_ds_* is the surface deposition velocity:
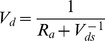
(11)


Furthermore, *R_a_* was calculated using the following equation, which is based on Erisman and Draaijers (1995) [25]. u* is the friction velocity, and *Ψ_h_* is the integrated stability function for heat.
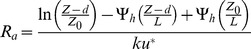
(12)


Next, the Wesely and Hicks (1977) model was used for more complex parameters, where u is the wind speed, *Sc* is the Schmidt number, and *Pr* is the Prandtl number:
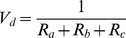
(13)




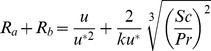
(14)




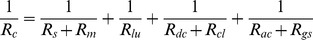
(15)


The resistances in the upper canopy included *R_s_* (the stomatal), *R_m_* (the mesophyll), and *R_lu_* (the outer surface resistances). The resistances in the lower canopy were the resistance to transfer by buoyant convection (*R_dc_*) and the resistance to the uptake by exposed surfaces (*R_cl_*). The fourth term represents resistances to transfer (*R_ac_*) and uptake (*R_gs_*) on the ground [26].

The third value, *V_d_*, was initially presented by Wesely et al. (1985) and was fit to the grassland ecosystem in the United States. This value can be calculated using equations 16 and 17. The first equation was fit to the stability condition (*L*>0), and the second equation was fit to the instability condition (*L*<0).
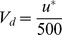
(16)




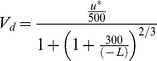
(17)


The third model was proposed by Ruijgrok and was fit to a European forest. If *V_s_* is the deposition velocity due to sedimentation, then *V_ds_* is calculated using the following equation:
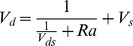
(18)




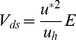
(19)




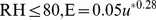
(20)




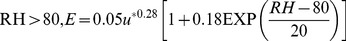
(21)




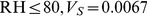
(22)




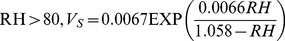
(23)


In the above equation, *RH* is the relative humidity, and *u_h_* is the wind speed at the top of the canopy (approximately 9 m).

## Results and Discussion

### 1. Atmospheric Conditions

**Figure 3:**
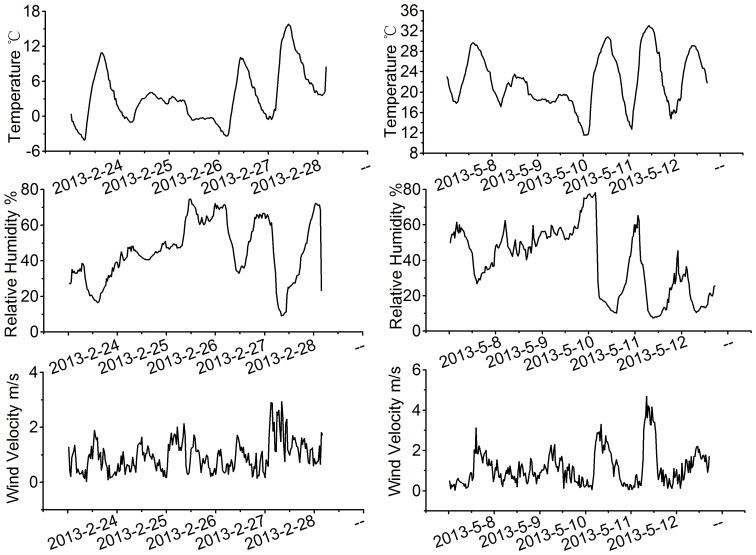
The variations of temperature, relative humidity and wind velocity at the study sites.

Meteorological conditions greatly impact the concentration of PM2.5 [27]. Therefore; therefore, the impacts of the conditions are important to analyze. Table 2 summarizes the meteorological conditions of Jiufeng National Forest Park and Olympic Forest Park, including temperature, humidity, wind speed, and solar radiation. Figure 3 presents the relationships between the meteorological conditions. We observed several common patterns between Jiufeng National Forest Park and Olympic Forest Park. The humidity and temperature were higher in the Olympic Forest Park, but the concentration of PM2.5, the solar radiation, and the wind speed were lower.

**Table 2:** The average values of, wind speed, temperature, humidity and solar radiation.

	Jiufeng National Forest Park				Olympic Forest Park			
	February		May		February		May	
	Daytime	Nighttime	Daytime	Nighttime	Daytime	Nighttime	Daytime	Nighttime
wind speed (m/s)	1.12±0.5	0.86±0.43	1.28±0.73	0.96±0.51	0.97±0.49	0.7±0.38	1.06±0.64	0.79±0.49
temperature (°C)	4.16±4.23	1.38±2.78	18.12±4.23	20.96±4.97	4.58±4.98	1.73±2.99	20.04±4.57	23.81±5.23
humidity (%)	44±17	50±19	56±21	62±23	42±18	47±18	53±20	59±22
solar radiation (w)	221±187	−1.24±1.3	343±231	1.21±1.6	208±199	−1.63±1.4	312±251	0.94±1.6

Wind speed, temperature, humidity and solar radiation in Jiufeng National Forest Park and Olympic Forest Park in February and May.

### 2. PM2.5 Gradients

Testing the changes in aerosol concentrations at multiple altitudes is difficult. The differences between the altitudes were minimal. A paired t-test reveals that the concentration at 15 m was significantly (P<0.01) higher than the concentration at 9 m during the spring (vegetation with young leaves), whereas the concentrations were not significantly different in the winter (vegetation without leaves). To decrease the sampling error, we used the same instrument to measure the concentration seven times at the same height of 9 m (or 15 m).

**Figure 4:**
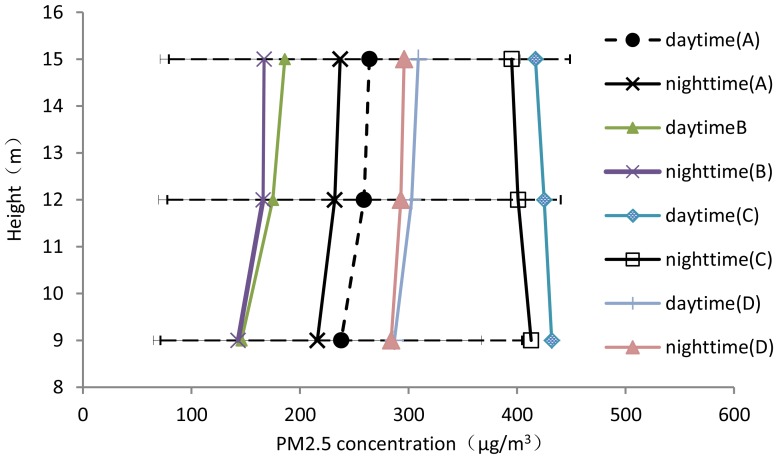
The mean PM2.5 concentration at the various heights and sites. The mean PM2.5 concentration at the heights of 9(A: Olympic Forest Park in spring. B: Jiufeng National Forest Park in spring. C: Olympic Forest Park in winter D: Jiufeng National Forest Park in winter).

Fig. 4 shows the average concentrations measured at the various heights, sites and times. The concentration at 15 m was greater than that at 9 m, and concentrations appeared to decrease with height. This observation indicates a downward flux of the particulate aerosols (top down). The results indicate that the forest may have had a high absorption ability. As demonstrated in Fig. 4, during the daytime or nighttime, the average concentrations in Jiufeng National Forest Park in February significantly decreased with height, regardless of the time of day. This finding could be primarily attributable to the *Platycladus orientalis*, that is a type of evergreen species. In contrast, the gradients indicated no significant change during the daytime and nighttime in Olympic Forest Park, that could be primarily attributable to the presence and nature of *Populus tomentosa* (a type of winter-deciduous tree). In conclusion, these results indicate that the evergreen forest had a higher absorption ability than the winter-deciduous forest in February. Similar results were obtained in May. These results may be because the *Platycladus orientalis* leaf structure is more complex than that of *Populus tomentosa*. The more complex the leaf structure, the more conducive the leaf is to adsorbing PM2.5.

### 3. The Concentration Ratios of PM2.5 from PM10

The concentration ratios of PM2.5 and PM2.5–10 (PM10 minus PM2.5) to PM10 reflect different sources of pollution among regions and seasons. In the winter, natural gas heating is used throughout Beijing instead of coal; however, there is a lower socioeconomic belt around Beijing that uses old-fashioned fireplace burning coal for heating [28]–[29]. This population’s reliance on coal heating is also responsible for the excessive sulfur dioxide discharge [30]–[31]. Another important source of pollution is the motor vehicle exhaust from the 5.3 million cars in Beijing [32].

**Figure 5:**
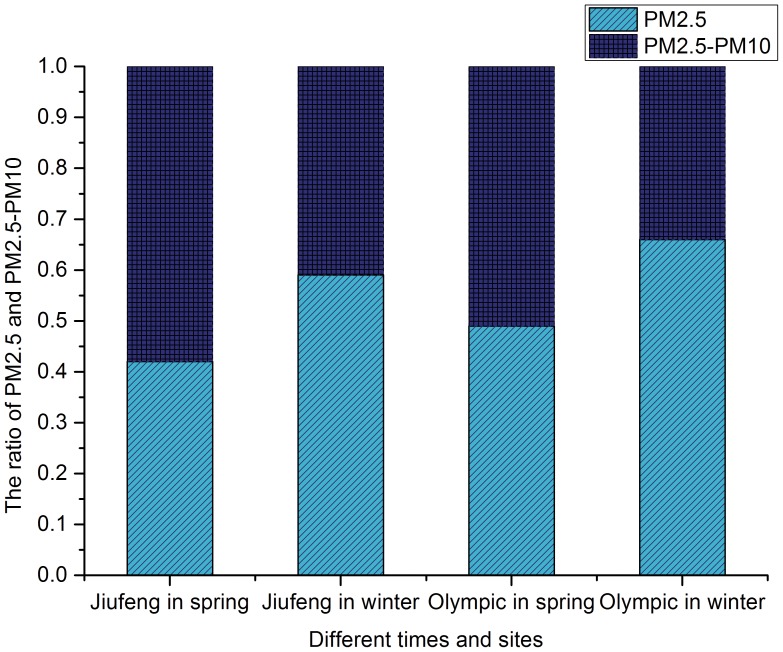
The concentration ratios of PM2.5 from PM10 at the study sites.

As shown in Fig. 5, the concentration ratios of PM2.5 to PM10 were 42% in spring and 59% in winter in Jiufeng National Forest Park and 49% in the spring and 66% in the winter in Olympic Forest Park. This indicates that the environmental conditions had an enormous impact on the concentration ratios of PM2.5.

### 4. The Change in the PM2.5 Concentration

Fig. 4 illustrates the diurnal variation of the PM2.5 concentrations in various seasons. PM2.5 concentrations in the winter were much higher than those in the spring in Jiufeng National Forest Park and Olympic Forest Park. The difference is due to the lack of leaves on the trees and the old-fashioned fireplaces (burning coal) in the areas circumjacent to Beijing, even though Beijing is a central heating city that uses gas [33]. Coal is the primary fuel for household heating and industrial production;burning coal produces more sulfur dioxide than burning oil or gas. As shown in Figure 4, two diurnal peaks occurred in the PM2.5 concentrations at the test sites: 6∶00–10∶00 and 18∶00–22∶00. This phenomenon may be due to two major sources of PM2.5 those times. The major sources of PM2.5 were automobile exhaust and coal burning. Measurements indicated that during the morning and afternoon rush hours, the mean daily traffic flow of Fifth Ring Road in Beijing was 200 vehicles per minute; occasionally, the traffic jams occurred [34].

These values far exceed the PM2.5 standard set by the national AAQS of China [35]. PM2.5 emissions increased during heater usage and when meteorological conditions were unfavorable for atmospheric dispersion.In other studies [36]–[37], lower PM2.5 concentrations were observed at special times, such as the 50th National Day in 1999, which can be attributed to specific procedures mandated by the government to reduce the emissions of PM2.5. Artificial simulation of rainfall was applied in the Che Gong Zhuang area on September 30, 1999. As a result, the PM2.5 concentration was notably low (50 µg/m^3^). From October 3 to 12, the weather conditions were characterized either by still air or calm wind, and the PM2.5 concentration increased two-fold and reached approximately 160 µg/m^3^ [36].

The PM2.5 concentrations gradually decreased from the winter to spring. The average PM2.5 concentration was 60% to 100% higher in the winter than in the spring, and 30% to 40% higher than the annual average [37] and was far greater than that of the EPA Ambient Air Quality Standard [38].

### 5. Deposition Velocities

**Figure 6:**
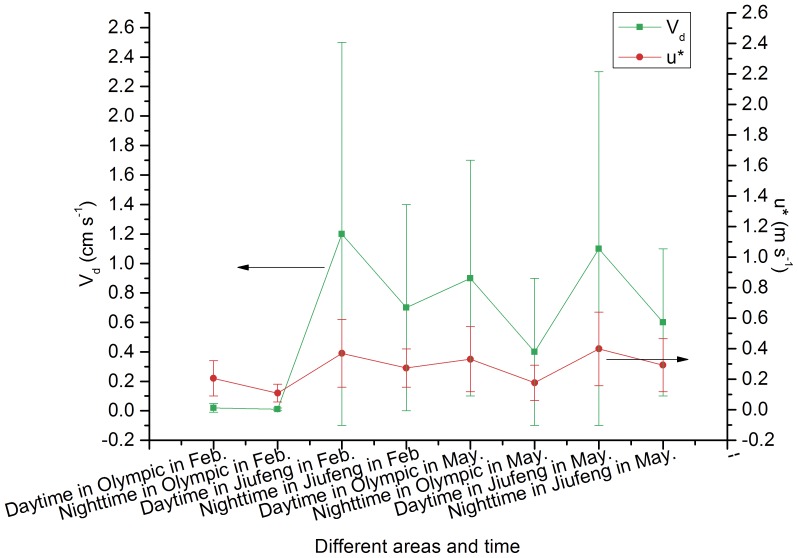
The relationship between u* and V_d_.

Table 3 lists the deposition velocities and relative parameters such as deposition velocity *V_d_* (cm s^−1^), friction velocity u* (m s^−1^), and Monine-Obukhov length *L* (m), during the daytime and nighttime, in Olympic Forest Park and Jiufeng National Forest Park. A positive correlation exists between u* and *V_d_* (Figure 6). The deposition velocities during the day and night in Olympic Forest Park were notably small in February because the vegetation in the forest lacked leaves. During February and May, the deposition velocities in Jiufeng were higher than in Olympic Forest Park. The main reason for this observation is that the PM2.5 capture rate of needle-leaved evergreen forests is higher than that of broadleaved deciduous forests [39].

**Table 3:** Mean deposition velocities, friction velocity and Monine-Obukhov length.

	V_d_ (cm s^−1^)	u* (m s^−1^)	L (m)
Daytime in Olympic in Feb.	0.02±0.03	0.22±0.12	−11.6±5.6
Nighttime in Olympic in Feb.	0.01±0.01	0.12±0.06	21.77±12.5
Daytime in Jiufeng in Feb.	1.2±1.3	0.39±0.23	−15.5±7.4
Nighttime in Jiufeng in Feb.	0.7±0.7	0.29±0.13	20.6±12.8
Daytime in Olympic in May.	0.9±0.8	0.35±0.22	−17.15±9.7
Nighttime in Olympic in May.	0.4±0.5	0.19±0.12	27.97±14.3
Daytime in Jiufeng in May.	1.1±1.2	0.42±0.25	−15.9±7.8
Nighttime in Jiufeng in May.	0.6±0.5	0.31±0.18	22.8±6.4

u* and L in the daytime and nighttime during the study period (February and May 2013). All of the values are the mean±standard deviation.

This study revealed that deposition increased in the daytime, whereas it decreased in the nighttime in Olympic Forest Park and Jiufeng National Forest Park. This result is consistent with previous studies. In Norway, Netherlands, America, Canada, Portugal [40], and Japan [10], the deposition velocities of aerosol particles were measured at the tops of coniferous forests. The measured values were greater than the computations by the Ruijgrok et al. (1997) model [23] in Kazuhide’s study [3]. However, the deposition velocities observed in this study were comparable to those reported in Kazuhide’s study [3]. Researchers from Japan suggested that the reason for this ambiguity could be a sampling error, as the measured values were larger than the modeled values. However, Horváth et al. [41] believed that it was important to correct the model parameters. Estimated *V_d_* and deposition flux were strongly influenced by eddy diffusivity in the roughness sub-layer [3]. The modification involves a height-dependent correction factor that ranges from 0.73 for *Z* = 22 m to 0.9 for *Z* = 34 m. In this study we used 0.64 [21], [42] as a correction factor for the calculations. Therefore, the deposition flux increased 56% during the day and increased 52% during the night. The increase in deposition flux during the day was greater than that at night. The same general patterns were observed with the deposition velocity.

### 6. Comparison between the Measured and Parameterized *V_d_*

**Figure 7:**
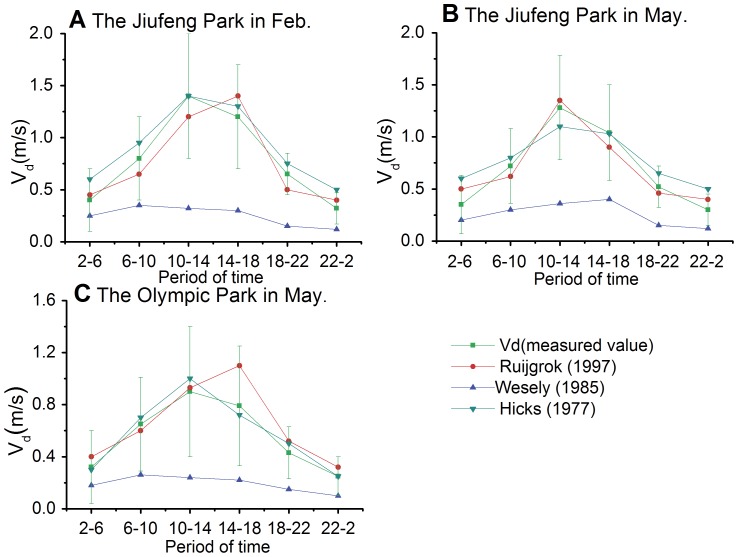
The trend of the deposition velocities at the study sites and during the study periods compared with other models. A: Jiufeng Park in February. B: Jiufeng Park in May. C: Olympic Park in May.

Because this type of data are not available for the Chinese mainland, it is very important to estimate the atmospheric deposition in this area. Figure 7. display the various deposition velocities at the study sites. The deposition velocities were calculated using the three models, and the error bars indicate the sampling errors in the experiment. We observed high deposition velocities during the daytime and low deposition velocities during the nighttime in Jiufeng National Forest Park and Olympic Forest Parks. The high deposition velocities in Jiufeng Park and the low deposition velocities in Olympic Park are consistent with the calculated results. However, the reproducibility of the Ruijgrok et al. (1997) model was relatively better than that of the Wesely et al. (1985) model. The calculated results from the Wesely et al. (1985) model were lower than those of the measured values.

In the summer, Kazuhide et al. [3] measured the deposition velocity of the PM2.5 sulfate in a deciduous forest at the eastern foot of Mt. Asama, Nagano Prefecture, central Japan using the gradient method. Kazuhide et al. [3] explained the methodology and asserted that the botanical structure factor (*LAI*) was not an accurate parameterization.

The measured value was higher than the calculated values from the Ruijgrok et al. (1997) model, the Hicks (1977) model and the Wesely et al. (1985) model. However, the Ruijgrok et al. (1997) model and the Hicks (1977) model were more suitable for Chinese forests and could better reflect the influence of the forest on PM2.5 deposition.

## Conclusion

The present study was conducted in Jiufeng National Forest Park and Olympic Forest Park in Beijing, China. To the best of our knowledge, this is the first report of the measurement of the deposition velocity of PM2.5 in China. This study also represents the first attempt to compare the deposition velocity in the city center with that in the suburbs.

In general, the deposition was higher during the daytime than during the nighttime. Likewise, the deposition was higher in the suburbs compared with the urban area. The deposition velocities of the aerosol particles were significantly higher in Jiufeng National Forest Park than in Olympic Forest Park during the same time periods. This may be due to the greater friction velocity in Jiufeng National Forest Park. Furthermore, the deposition velocities of the aerosol particles were also influenced by the eddy diffusion coefficient of the sub-layer surface roughness, as indicated by the report of Kazuhide et al [3].

Our results indicated that the deposition velocities of the aerosol particles were influenced by the friction velocity (Figure 6). The friction velocity was strongly influenced by the aerodynamic conditions [22], [23], [24], [42]. Thus, the deposition velocities of the aerosol particles were strongly affected by aerodynamic conditions. These results are consistent with most recently reported results from other centers [22]–[24].

A potential sampling error resulted in arger measured values compared with the model values. Therefore, it was important to correct the model parameters to calculate the deposition velocities of PM2.5 and to compare the calculated values of the models with the measured values.

The measured results were more consistent with the Ruijgrok et al. (1997) model than with the Wesely et al. (1985) model. Different models were applicable to different profiles of the forests in different regions. The Ruijgrok et al. (1997) model was more applicable to the coniferous and broadleaved forests in northern China.

## Supporting Information

Data S1 **Data S1 is the data of meteorological data and PM2.5 concentration.** This data contain seven parts: 1. meteorological data1 include temperature,humidity,wind direction,wind speed et al in different height in Olympic Forest Park. 2. meteorological data2 include temperature,humidity,wind direction,wind speed et al in different height in Jiufeng National Forest Park. 3. met data include Monin-Obucov length and friction velocity. 4. PM2.5 DATA1 is the PM2.5 concentration in winter of Jiufeng National Forest Park. 5. PM2.5 DATA2 is the PM2.5 concentration in spring of Olympic Forest Park. 6. PM2.5 DATA3 is the PM2.5 concentration in winter of Olympic Forest Park. 7. PM2.5 DATA4 is the PM2.5 concentration in spring of Jiufeng National Forest Park.
(XLS)

## References

[1] Schindlbacher A, Zechmeister-Boltenstern S, Butterbach-Bahl K (2004) Effects of soil moisture and temperature on NO, NO_2_, and N_2_O emissions from European forest soils. J Geophys Res 109: D17302.

[2] Williams E, Guenther A, Fehsenfeld F (1992) An inventory of nitric oxide emissions from soils in the United States. J Geophys Res 97: 7511–7519.

[3] Kazuhide M, Yoshifumi F, Kentaro H (2010) Deposition velocity of PM2.5 sulfate in the summer above a deciduous forest in central Japan. Atmos Environs 44, 4582–4587.

[4] Parkin CS (1987) Factors affecting the movement of spray droplets above a forest canopy. In: Proceedings of Symposium on the aerial application of pesticides in forestry, National Research Council 69–79.

[5] Grace J (1983) Plant-atmosphere relationships. London, New York: Chapman and Hall.

[6] Elshout AJ, Viljeen JW, van Duuren H (1978) Sulphates and sulphuric acid in the atmosphere in the years 1971–1976 in the Netherlands. Atmos Environ 12: 785–790.

[7] Matsuda K, Takahashi A, Hayashi K, Sorimachi A (2007) A review of field studies on dry deposition in East Asia. Journal of Japan Society for Atmospheric Environment 42, 261–270 (in Japanese).

[8] Takahashi A, Sato K, Wakamatsu T, Fujita S, Yoshikawa K (2002) Estimation of dry deposition of sulfur to a forest using an inferential method enfluence of canopy wetness on SO_2_ dry deposition. Journal of Japan Society for Atmospheric Environment 37: 192–205 (in Japanese).

[9] Matsuda K, Aoki M, Zhang S, Kominami T, Fukuyama T, et al (2002) Dry deposition velocity of sulfur dioxides on a red pine forest in Nagano, Japan. Journal of Japan Society for Atmospheric Environment 37: 387–392.

[10] Hayashi K, Takagi K, Noguchi I, Fukuzawa K, Takahashi H, et al (2009) Ammoniacal nitrogen emission from a young larch ecosystem afforested after clear-cutting of a pristine forest in northernmost Japan. Water Air Soil Poll 200: 33–46.

[11] Sorimachi A, Sakamoto K, Ishihara H, Fukuyama T, Utiyama M, et al (2003) Measurements of sulfur dioxide and ozone dry deposition over short vegetation in northern China-a preliminary study. Atmos Environ 37: 3157–3166.

[12] Sorimachi A, Sakamoto K, Sakai M, Ishihara H, Fukuyama T, et al (2004) Laboratory and field measurements of dry deposition of sulfur dioxide onto Chinese loess surfaces. Environ Sci Technol 38: 3396–3404. 15260340 10.1021/es034967p

[13] Tsai J, Chen C, Tsuang B, Kuo P, Tseng K, et al (2010) Observation of SO_2_ dry deposition velocity at a high elevation flux tower over an evergreen broadleaf forest in Central Taiwan. Atmos Environ 44: 1011–1019.

[14] Matsuda K, Watanabe I, Wingpud V, Theramongkol P, Khummongkol P, et al (2005) Ozone dry deposition above a tropical forest in the dry season in northern Thailand. Atmos Environ 39: 2571–2577.

[15] Matsuda K, Watanabe I, Wingpud V, Theramongkol P, Ohizumi T (2006) Deposition velocity of O_3_ and SO_2_ in the dry and wet season above a tropical forest in northern Thailand. Atmos Environ 40: 7557–7564.

[16] Chen JR (2013) We should do a good job on further improving coal efficiently and cleanly utilization. China Energy 35: 5–8.

[17] Xu J, Ding GA, Yan P, Zhang JC, Wang SF (2006) Effect of firecracker setting off on the fine particle pollution in Beijing downtown areas. Journal of Safety and Environment 6: 79–82.

[18] Businger JA (1986) Evaluation of the accuracy with which dry deposition can be measured with current micrometeorological techniques. Journal of Climate and Applied Meteorology 25: 1100–1124.

[19] Erisman JW, Draaijers GPJ (1995) Atmospheric Deposition in Relation to Acidification and Eutrophication, Studies in Environmental Science, vol. 63. ELSEVIER, 55–75.

[20] Wyers GP, Duyzer JH (1997) Micrometeorological measurement of the dry deposition flux of sulphate and nitrate aerosols to coniferous forest. Atmos Environ 31: 333–343.

[21] Wesely ML, Hicks BB (1977) Some factors that affect the deposition rates of sulfur dioxide and similar gases on vegetation. Journal of the Air Pollution Control Association 27: 1110–1116.

[22] Wesely ML, Cook DR, Hart RL (1985) Measurement and parameterization of particulate sulfur dry deposition over grass. J Geophys Res 90: 2131–2143.

[23] Ruijgrok W, Tieben H, Eisinga P (1997) The dry deposition of particles to a forest canopy: a comparison of model and experimental results. Atmos Environ 31: 399–415.

[24] Slinn WGN (1982) Predictions for particle deposition to vegetative canopies. Atmos Environ 16: 1785–1794.

[25] Erisman JW, Draaijers G, Duyzer J, Hofshreuder P, van Leeuwen NFM, et al (1997) Particle deposition to forests-summary of results and application. Atmos Environ 31: 321–332.

[26] Lamaud E, Carrara A, Brunet Y, Lopez A, Druilhet A (2002) Ozone fluxes above and within a pine forest canopy in dry and wet conditions. Atmos Environ 36: 77–88.

[27] Amos PK, Loretta JM, Daniel JJ (2010) Correlations between fine particulate matter (PM2.5) and meteorological variables in the United States: Implications for the sensitivity of PM2.5 to climate change. Atmos Environ32: 3976–3984.

[28] Guo P, Lai KP (2009) Study on Regional Energy Use in the Farmer’s Life in China. Journal of Anhui Agri. Sci. 37(23): 11236–11237 (in Chinese)..

[29] Ministry of Agriculture of the People’s Republic of China (2006) Statistical Data of National Rural Renewable Energy [R], 2007 (in Chinese).

[30] National Bureau of Statistics of China (2007) China Energy Statistical Yearbook[M]. Beijing: China Statistics Press (in Chinese).

[31] Zhou ZR, Wu WL, Wang XH (2009) Analysis of Changes in the Structure of Rural Household Energy Consumption in Northern China: A Case Study[J]. Renewable& Sustainable Energy Reviews 13(1) 176–182 (in Chinese)..

[32] Beijing Transportation Research Center (2006) Beijing Transport Annual Report [M]. Beijing: Beijing Transportation Research Center: 32–35 (in Chinese).

[33] Beijing Development and Reforming Office (2012) Beijing twelve-Five formulation of energy planning[M]. Beijing: Beijing Development and Reforming Office (in Chinese).

[34] Yang HM, Wang HZ, Wu YB (2011) Observation and Characteristics Analysis of Traffic Flow in Nanjing. Environmental Science and Technology of China. 24(2): 98–101 (in Chinese)..

[35] US EPA (2008) National ambient air quality standards (NAAQS) [EB/OL] Washington DC:US EPA, Office of Air Quality Planning and Standards. [2013–02–22]. http:www.epa.gov/air/criteria.html.

[36] He KB, Ma YL, Zhang Q, Yu XC, Yang FM (2002) Variation characteristics of PM2.5 concentration and its relationship with PM10 and TSP in Beijing. J Environ Sci-China 22(6): 506–510.

[37] Yang FM, He KB, Ma YL (2002) Chemical characteristics of PM2.5 species in Beijing ambient air. J Tsinghua Univ (Sci. &Tech. 42 12: 1605–1608.

[38] The state Department of Environmental Protection (2012) GB3095–2012 Ambient air quality standards[S]. Beijing: China Environmental Science Press.

[39] Cheng ZH, Wu JY, Liu YG, Li HZ, Xiong YS, et al (2004) Effects of Main A forestation Tree Species on Dust Blocking in Yueyang City. Journal of Chinese urban forestry 2(2): 37–40.

[40] Feliciano MS, Pio CA, Vermeulen AT (2001) Evaluation of SO_2_ dry deposition over short vegetation in Portugal. Atmos Environ 35: 3633–3643.

[41] Horváth L (2003) Dry deposition velocity of PM2.5 ammonium sulfate particles to a Norway spruce forest on the basis of S- and N-balance estimations. Atmos Environ 37: 4419–4424.

[42] Duyzer JH, Weststrate JH (1995) The use of the gradient method to monitor trace gas fluxes over forest: flux-profile functions for ozone and heat. In Acid Rain Research: Do We Have Enough Answers? (edited by Heij G. J. and Erisman J. W.), 21–30. Elsevier, Amsterdam.

